# Insights into the Enhanced Ceftazidime Hydrolysis by Ent385 AmpC β‑Lactamase from Multiscale Simulations

**DOI:** 10.1021/acscatal.5c02383

**Published:** 2025-06-23

**Authors:** Anderson H. Lima, Marc W. van der Kamp

**Affiliations:** † School of Biochemistry, University of Bristol, University Walk, Bristol BS8 1TD, U.K.; ‡ Laboratório de Planejamento e Desenvolvimento de Fármacos, Instituto de Ciências Exatas e Naturais, Universidade Federal do Pará, Rua Augusto Corrêa, 01, 66075-110, Belém, Pará, Brasil

**Keywords:** QM/MM simulations, antibiotic resistance, ceftazidime hydrolysis, Enterobacter cloacae, transition state analysis

## Abstract

The emergence of multidrug-resistant bacteria poses a significant threat to public health. Particularly, they are becoming increasingly resistant to β-lactam antibiotics, which are one of the most important drug classes for the treatment of bacterial infections. Ceftazidime-avibactam has shown promising activity against highly drug-resistant bacteria, including carbapenem-resistant Enterobacterales. However, an Ala294-Pro295 deletion in the Class CAmpC β-lactamase can confer reduced susceptibility to these agents. In this study, we investigated the molecular mechanisms underlying the enhanced hydrolysis of ceftazidime by Ent385 AmpC β-lactamase with the deletion using quantum mechanics/molecular mechanics (QM/MM) simulations. We used constant pH molecular dynamics simulations of the β-lactamase-ceftazidime acyl-enzyme complex to verify the likely protonation states, confirming Tyr150 primarily exists as a tyrosinate. We then used QM/MM (DFTB2/ff14SB) umbrella sampling to calculate the reaction-free energy barriers (Δ^‡^ *G*) for the deacylation step of cephalosporin hydrolysis. This reveals that Tyr150 (rather than the substrate) acts as the base. Importantly, the difference in Δ^‡^ *G* between the canonical AmpC (P99) and the Ent385 variant with Ala294-Pro295 reinserted, on the one hand, and the Ent385 variant, on the other, was in very good agreement with the difference deduced from experimental kinetic data. Detailed analysis of the transition state ensembles, alongside additional simulations, shows that the Ala294-Pro295 deletion allows the entrance of an additional water molecule that helps stabilize the tetrahedral intermediate. Overall, our QM/MM simulations provide valuable insights into the reaction mechanism and reasons for enhanced ceftazidime breakdown. The protocol used in this study successfully captures the kinetic differences observed among the studied variants. This approach can be employed to investigate other Class C β-lactamase variants with similar features, providing insights into their mechanisms and potential contributions to reduced susceptibility to antibiotic treatments.

## Graphical abstract

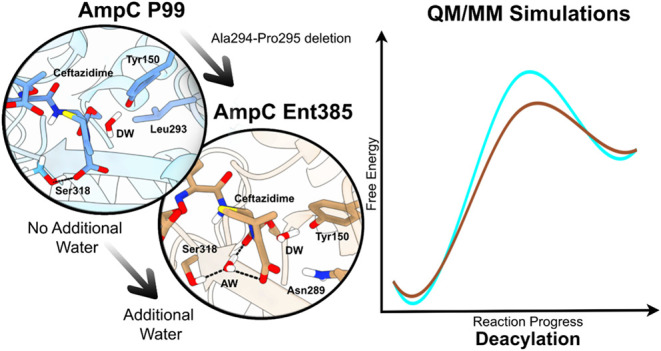

## Introduction

The increase in bacteria developing resistance to multiple drugs is becoming a significant global health concern, threatening the effectiveness of conventional treatments. A particular concern is the growing resistance these pathogens are exhibiting against β-lactam antibiotics.^1^ These drugs have been a strong defense against bacterial infections, yet their efficacy is increasingly threatened by the proliferation of resistance mechanisms, in particular, those mediated by β-lactamases.^2^ Over the past decades, combination therapy utilizing both a β-lactam antibiotic and a β-lactamase inhibitor has proven to be effective in managing resistance for the most difficult-to-treat infections. In particular, the combination of ceftazidime-avibactam (FDA approved in 2015) has emerged as a promising solution, with both classified as “critically important” by the WHO.^3^ This combination exhibits strong activity against highly resistant bacteria, including those resistant to carbapenems.^4,5^ However, the identification of variants of different β-lactamases has raised uncertainties regarding the future effectiveness of this treatment strategy. One particular case is the Ala294-Pro295 deletion found in the R2 loop of ’s AmpC Ent385 β-lactamase, which increases the efficiency in hydrolyzing ceftazidime over 1000 fold, compared to AmpC without this deletion, as found in AmpC P99.^6^

β-lactamases can be classified into four classes, A, B, C, and D (Ambler classification), according to their sequence similarity and catalytic mechanism of action.^7,8^ Classes A, C, and D function by a serine ester hydrolysis mechanism, whereas class B β-lactamases have zinc ions participating in catalysis.^8^ Compared to the class A and D serine β-lactamases, the detailed hydrolysis mechanism of class C β-lactamases, also known as AmpC, has remained unclear.^2^ For the initial acylation step, Lys67 is believed to act as the general base to facilitate the nucleophilic attack by Ser64 on the carbonyl carbon of the β-lactam ring.^9^ The proposed mechanism for deacylation, on the contrary, is extensively debated.^2,10^ Notably, for hydrolysis of ceftazidime by AmpC β-lactamases, multiple studies indicate that this deacylation step is kinetically decisive.^11–13^ There are those suggesting that the β-lactam ring nitrogen is involved in the process (substrate-activated), and those proposing that proton transfers occur between Lys67, Tyr150, and the deacylating water (DW) without direct participation of the substrate (conjugate base); see Figure 1.

**Figure 1:**
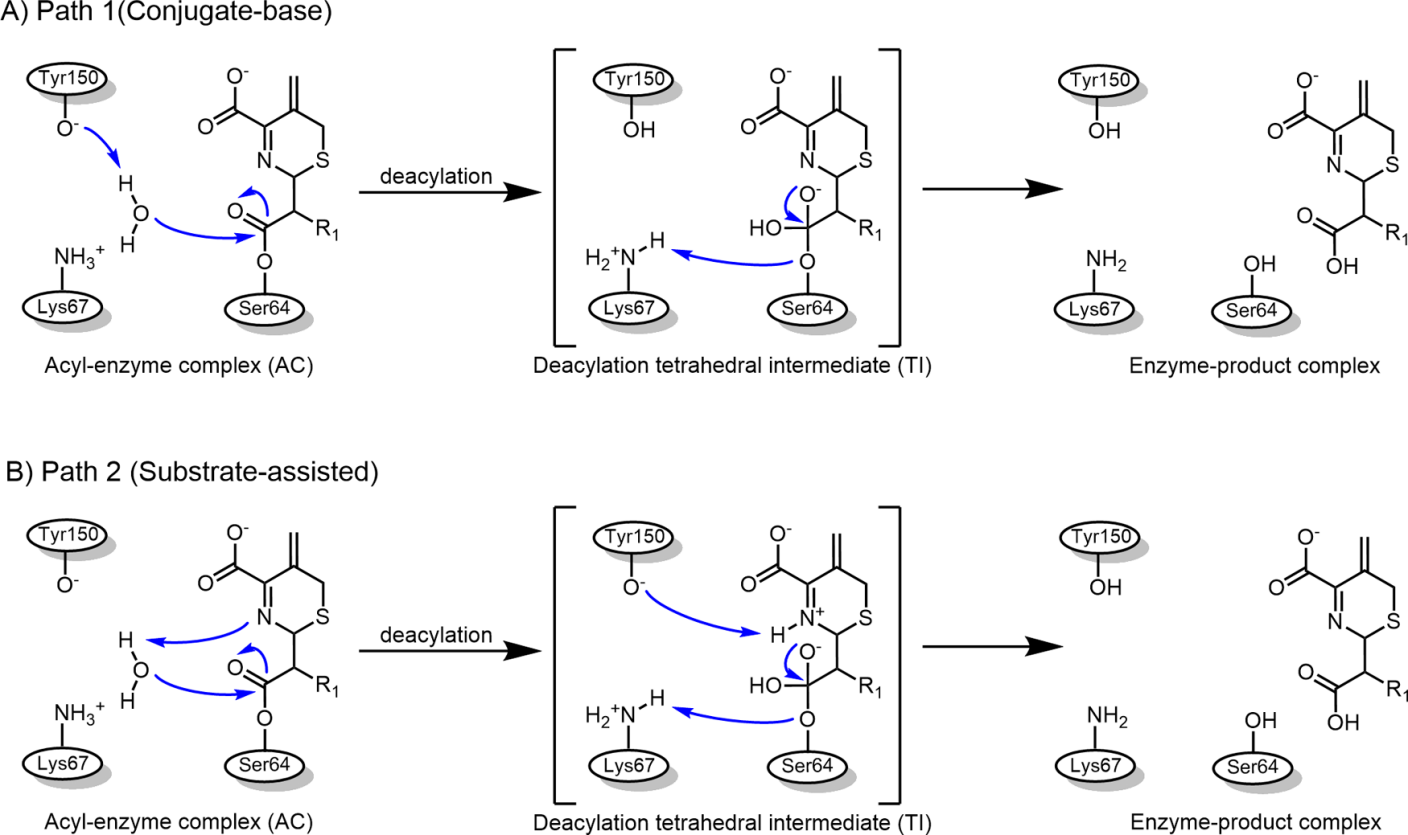
Possible mechanisms of acyl-enzyme deacylation in class C β-lactamases. (A) Tyr150 (as tyrosinate) acting as “conjugate base” to deprotonate the deacylating water (Path 1). (B) “Substrate-assisted” mechanism, with substrate N deprotonating the deacylating water (Path 2).

Tripathi and Nair^14^ used QM/MM simulations to indicate that deprotonation of Tyr150 by transferring its proton to Lys67 is a critical step occurring prior to the activation of the DW molecule in the deacylation of the good substrate cephalotin. Thus, Tyr150 may function as the general base, while Lys67 can subsequently act as a general acid, donating a proton to Ser64, which facilitates the regeneration of the free enzyme and the release of the hydrolyzed product.^15,16^

The alternative suggestion that the nitrogen in the β-lactam ring can act as the base (“substrate-assisted”) is supported by, for example, the high-resolution crystal structure of a tetrahedral intermediate model^17^ and the observation that the absence or presence of the nitrogen can distinguish β-lactam inhibitors (i.e., no deacylation) from β-lactam substrates.^18^ This distinction differentiates β-lactam antibiotics that are effective against AmpC-expressing bacteria from those to which these pathogens are resistant.^19^ Thus, it has been suggested that this substrate nitrogen could act as a base, abstracting a proton from the DW. As a result, the mechanism of β-lactam hydrolysis in class C enzymes has remained unclear, and it is possible that either the “conjugate base” or “substrate-assisted” mechanism may dominate, depending on the enzyme–substrate combination.^2^

Here, we focused on deacylation of ceftazidime, with the main aim to unravel the molecular mechanism underlying the enhanced hydrolysis of ceftazidime by Ent385 AmpC β-lactamase, using quantum mechanics/molecular mechanics (QM/MM) molecular dynamics simulations of the reaction. First, we sought to determine the most likely deacylation mechanism for this important substrate, specifically identifying which base abstracts a proton from the DW during the ceftazidime hydrolysis process. Subsequently, we investigated the reasons behind the enhanced activity of Ent385 in comparison to the canonical P99 AmpC β-lactamase and the Ent385_Rev variant, which is characterized by the reversion of the alanine-proline deletion at positions 294–295 in the R2 loop. Through our QM/MM simulations, we provide mechanistic details that explain experimentally determined differences in kinetics and thereby shed light on the origins of enhanced ceftazidime hydrolysis by the Ent385 AmpC β-lactamase.

## Methods

### Modeling Acyl-Enzyme Structures

The starting structure of AmpC Ent385 in complex with ceftazidime (acyl enzyme) was taken from the crystal structure of this complex, Protein Data Bank (PDB) code 6LC9.^6^ The coordinates for AmpC P99 were taken from PDB code4XUX­(complex with a covalent boronic acid inhibitor, RPX-7009),^20^ and ceftazidime was modeled in through alignment with 6LC9 (no clashes with protein residues occur). To obtain a model of the Ent385_Rev variant (in which Ala294-Pro295 deletion was reverted),^6^ an AlphaFold 3 (AF3)^21^ model structure was used. The region spanning residues Lys280-Pro296, which is hypothesized to adopt a distinct conformation in the variant, was extracted from the AF3 model. This segment was then aligned to the corresponding region of the AmpC Ent385-ceftazidime complex (PDB 6LC9) and subsequently integrated into this structure, replacing the original loop. No steric clashes between the inserted AF3-derived segment and the rest of the protein or ligand were observed.

The parameters used for the ceftazidime adduct with Ser64 were the same as those described previously.^22^ The AmpC Ent385 PDB file was used as the starting structure for constant pH MD (CpHMD) simulations performed in both implicit and explicit solvent. In the implicit solvent simulations, Generalized Born implicit solvent (using the GB^OBC^ model, igb = 2) was employed with the modified Amber ff10 force field and appropriate PBRadii set (mbondi2).^23^ For the explicit solvent simulations, the Amber ff14SB force field^24^ was used in conjunction with TIP3P water^25^ to treat the aqueous environment. In these CpHMD simulations, the catalytic residues Lys67, Tyr150, and Lys315 were titrated and their protonation and deprotonation events were evaluated during simulations at a pH of 7.2. All CpHMD simulations were carried out with the Amber20 program^26^ (See details in SI).

The protonation states (including histidine tautomers) of the other residues were estimated using the H++ web server.^27^ His198 His186, His193, and His313 were protonated on NE2. His39 and His210 are protonated on both ND1 and NE2. The corresponding histidines in AmpC P99 are protonated accordingly: His39 and His186 (both ND1 and NE2), His198 and His313 (ND1). All other titratable residues are in their standard protonation states. All starting structures and parameters are available as Supporting Information.

After determining the relevant protonation states of the titratable residues at pH 7.2 (used to determine steady-state kinetics by Kawai et al.),^6^ MM MD simulations in explicit solvent were performed of the ceftazidime acyl-enzymes (AC) of AmpC Ent385, Ent385_Rev, and P99. The systems were solvated in a cubic box with TIP3P^25^ water molecules, ensuring a minimum distance of 10 Å between the protein and the box edges. Four Cl^–^ ions were added to neutralize the system by randomly replacing bulk water molecules. The protein was described using ff14SB.^24^ For MM MD simulations, energy minimization, heating, and equilibration were performed (see SI for details). Production runs were performed in the NPT ensemble at 300 K and 1 atm using a 2 fs time step and the SHAKE algorithm. The default cutoff for direct-space nonbonded interactions was used, with Particle-Mesh Ewald used for long-range electrostatics. For each AC system, four independent simulations of 120 ns each were performed and analyzed using CPPTRAJ.^28^

### QM/MM Simulations of Ceftazidime Deacylation

For QM/MM MD simulations, the self-consistent charge density functional tight-binding (SCC-DFTB, DFTB2) semiempirical method^29,30^ was used to describe the energy of the QM region (using the standard mio-1–1 integral parameters) while the remainder of the system was described by ff14SB^24^ (protein, together with GAFF-based parameters for the ceftazidime adduct described previously^22^) and TIP3P (water). In these simulations, SHAKE was removed for the QM region only and a time step of 1 fs was used, with other settings identical to the MM MD simulations. A total of 70 atoms were included in the QM region (Figure S1), which consisted of the entire ceftazidime (CTZ), the Ser64 and Tyr^–^150 side chains (from Cβ), and the deacylating water (DW). The valences of the QM atoms at the QM/MM boundary were satisfied using the “link atom” method.^31^ The charge and spin multiplicity for the QM region were defined as −3 and 1, respectively. DFTB2/ff14SB was used successfully previously to distinguish deacylation differences in Class A β-lactamases.^32–34^

Two-dimensional umbrella sampling QM/MM MD simulations were performed at the DFTB2/ff14SB level to compare two different proposed mechanisms: “conjugate base” using Tyr150 (Path 1) and “substrate-activated” (Path 2), see Figure 1. Path 1 was followed starting from the AC complex using two Reaction Coordinates (RC_1_ and RC_2_) values to sample formation of the tetrahedral intermediate (TI). RC_1_ describes the proton transfer from DW, and RC_2_ describes the nucleophilic attack of DW on the acyl-enzyme carbonyl (RC_2_ = d­(O_DW_-C8_CTZ_)). In Path 1, RC_1_ refers to the proton transfer between DW and Tyr150 as follows: RC_1_ = d­(Oη_Tyr150‑_H_DW_) – d­(O_DW_-H_DW_). In Path 2, RC_1_ refers to proton transfer between the DW and the substrate: RC_1_ = d­(N5_CTZ_-H_DW_) – d­(O_DW_-H_DW_).

The RCs were harmonically restrained to values ranging from −1.0 to 1.0 Å in RC_1_ and from −3.4 to 1.4 Å in RC_2_, with step sizes of 0.1 Å and a force constant of 100 kcal mol^–1^ Å^–2^, and RC_2_ was incremented further, using previous windows as starting points to sample all RC values. Then, 20 ps QM/MM MD production was run, with RC distances recorded every fs to build the 2D free energy profile employing the Weighted Histogram Analysis Method (WHAM),^35^ using the wham2d code from the Grossfield lab,^36^ where 80 bins were used for each reaction coordinate. We performed at least two independent replicates of the QM/MM umbrella sampling simulations using an identical protocol for all of the evaluated systems. The final reported barrier values are the average of these independent runs, and the associated uncertainty is expressed as the standard deviation. Minimum free energy paths were determined using MEPSA v1.4.^37^ For the purpose of testing the accuracy of the DFTB2 method for the reaction, umbrella sampling was also run using the same protocol but with the QM region extended to include the whole Ser64 residue and adjacent backbone atoms (Figure S1). Finally, comparison to DFT was done by exploring the potential energy surface, employing DFTB2/ff14SB and M06–2X single-point energy calculations with a large QM region (Figure S2; see further Results and Discussion).

## Results and Discussion

### Tyr150 is Positioned to Act as a Base for Deacylation of the Ceftazidime Acyl-Enzyme Complex in AmpC Ent385 β-Lactamase

Throughout this work, we will use a standardized amino acid numbering scheme that was recently developed for class C β-lactamases,^38^ so that residue numbers of the key active site residues remain the same for AmpC P99 and AmpC Ent385 (despite the Ala294-Pro295 deletion in the latter, as shown in Figure S3). Before investigating the reaction, constant pH MD simulations in both implicit and explicit solvents were used to examine the likely protonation states of the catalytically important active site residues Lys67, Tyr150, and Lys315. The results shown in Table 1 were obtained with the ceftazidime acyl-enzyme complex of AmpC Ent385 (as taken from PDB ID 6LC9,^6^ resolution 1.65 Å).

**Table 1:** Prediction of Protonation States at pH 7.2 of Important Catalytic Residues in the AmpC Ent385 Acyl-Enzyme Complex with Ceftazidime from Constant pH MD Simulations

residue	predicted p*K*_a_ apo (H++)^27^	p*K*_a_ in implicit solvent CpHMD	p*K*_a_ in explicit solvent CpHMD	average fraction protonated (explicit solvent CpHMD)
Lys67	>12.0	7.7 ± 0.1	7.6 ± 0.4	0.73 ± 0.05
Tyr150	6.3	7.2 ± 0.3	7.1 ± 0.2	0.43 ± 0.13
Lys315	>12.0	∞	8.2 ± 0.4	0.95 ± 0.07

Considering the average of both implicit and explicit solvent CpHMD simulations at pH 7.2, our results show dynamic behavior in the protonation states of Lys67 and Tyr150, with multiple transitions observed in three independent runs. Lys67 remained predominantly protonated (∼68–78%), while Tyr150 exhibited greater variability, shifting between protonated and deprotonated forms, visiting each with approximately equal likelihood. In contrast, the states visited for the protonated Lys315 configuration account for approximately 95% occupancy, meaning that Lys315 is almost fully protonated. These protonation states are consistent with p*K*_a_ predictions obtained using H^+2^ ^27^ for the free enzyme (ceftazidime not included), see Table 1. These indicate that Tyr150 is predominantly deprotonated in the apo enzyme (predicted p*K*_a_ 6.3), while both Lys67 and Lys315 are protonated. The dynamics of proton exchange, particularly for the Lys67/Tyr150 pair, was previously investigated by Tripathi and Nair for the 908R class C β-lactamase, who indicated that, among the various proposed protonation states of the apo protein, the most thermodynamically and kinetically stable configuration is where Tyr150 is deprotonated, while Lys67 and Lys315 remain protonated.^39^ Their results showed that the stability of this protonation state is significantly affected by noncovalent binding of cephalothin. Here, a slight increase in the p*K*_a_ is found for Tyr150, with the protonated and deprotonated states present equally at pH 7.2, and a larger decrease in p*K*_a_ is found for Lys67. Tooke et al.^2^ emphasize the balance between the roles of Tyr150 and Lys67 in the acylation and deacylation steps. Although the deprotonation of Tyr150 by Lys67 is not a rate-limiting step, it has been considered critical for the deacylation reaction, occurring before the activation of the catalytic water molecule.^14^

Previous studies by Adediran and Pratt^40^ and Díaz et al.^9^ explored the possible protonation states of the Lys67/Tyr150 pair in class C β-lactamases. Our CpHMD simulations indicate that two protonation states are accessible (with similar likelihood) in the ceftazidime acyl-enzyme complex with AmpC Ent385. The protonation state Lys67^+^/Tyr150^–^/Lys315^+^ is referred to as the Tyrosinate configuration, while Lys67/Tyr150/Lys315^+^ is referred to as the Tyrosine configuration. We performed conventional MM MD simulations in explicit solvent on both configurations, showing that the protonation states of Lys67 and Tyr150 have a significant impact on the detailed active site conformation of the acyl-enzyme (AC) ensemble. The results suggest that the configuration with Tyr150 negatively charged is more favorable for deacylation compared with neutral Tyr150. In the former Tyrosinate configuration, a water molecule is more frequently located in the optimal position for deacylation: a higher density of water molecules is observed around the electrophilic carbon of ceftazidime, particularly within the 3.5–4.0 Å range (Figure S4). Hydrogen bond analysis for the AC complex of AmpC Ent385 with ceftazidime indicates that when Lys67 is neutral, it predominantly forms a hydrogen bond with Tyr112 (Figure S5). It has been reported that the orientation of Tyr150 can be influenced by hydrophobic interactions with the Tyr112 side chain, with correlated movement observed between these residues.^9^ Additionally, Lys315 was found to frequently interact with Tyr150, contributing to the stability of the tyrosinate. These results highlight the role of Lys67 in the deacylation process. When Lys67 is in its neutral state and accepts a hydrogen bond from Tyr112, it is not properly oriented to act as a base for abstracting a proton from the deacylating water during its nucleophilic attack. In contrast, with Lys67 protonated, Tyr150 (in its tyrosinate state) is well-positioned to fulfill this role, emphasizing the likely importance of a coordinated role of Lys67 and Tyr150 in the deacylation mechanism. Finally, after the formation of the deacylation tetrahedral intermediate, Lys67 (in its protonated form) can transfer a proton to Ser64, restoring the active site of AmpC Ent385 for subsequent hydrolysis cycles (Figure 1). Based on these observations, the Tyrosinate configuration can be considered the most relevant for deacylation activity and was thus chosen as the starting point for the subsequent QM/MM reaction simulations.

### Ceftazidime Deacylation in AmpC Involves Tyr150 Acting as a Base

We performed QM/MM (DFTB2/ff14SB) umbrella sampling simulations to compare the two different proposed ceftazidime deacylation mechanisms shown in Figure 1, in AmpC Ent385. The aim of this was to evaluate which base most likely abstracts the proton from the deacylating water (DW), and whether this deprotonation of DW is concerted with its nucleophilic attack on the acyl carbon. Reaction-free energy barriers (Δ^‡^ *G*) for the deacylation step of ceftazidime hydrolysis indicate that the “conjugate base” mechanism with Tyr150 acting as the base (Path 1) is favored by 7.2 kcal/mol over the “substrate-assisted” mechanism (Path 2). Thus, according to our simulations, Tyr150 acts as a base in the ceftazidime deacylating reaction. The minimum free energy pathway (Figures 2 and S2) shows that proton transfer and nucleophilic attack occur in a concerted fashion. Although the transition state is located approximately at the same point on the free energy surface for both Path1 and Path 2, it is observed that in Path1, the network of hydrogen bond interactions is more favorable for stabilizing the intermediates than in Path 2. During the TI formation in Path 1, the OH group added to the electrophilic carbon of ceftazidime forms a stabilizing hydrogen bond with the hydroxyl group of Tyr150, where Tyr150 acts as a hydrogen bond donor. In contrast, in Path 2, this OH group potentially serves as a third hydrogen bond donor to the oxyanion of Tyr150, which is already stabilized by interactions with Lys67 and Lys315. This difference in hydrogen bonding likely contributes to the increased stability of the TI and also the lower activation energy observed in Path 1 compared with Path 2. We further measured the distance between the oxyanion of Tyr150 and the protonated substrate nitrogen in the TI obtained from Path 2 (4.35 ± 0.19 Å). This relatively long distance suggests that regeneration of the active site of AmpC Ent385 may come at an additional energy cost. Specifically, Tyr150 would need to capture the proton from the nitrogen of ceftazidime, likely occurring after TI formation, either as the TI collapses or immediately following product formation.

**Figure 2:**
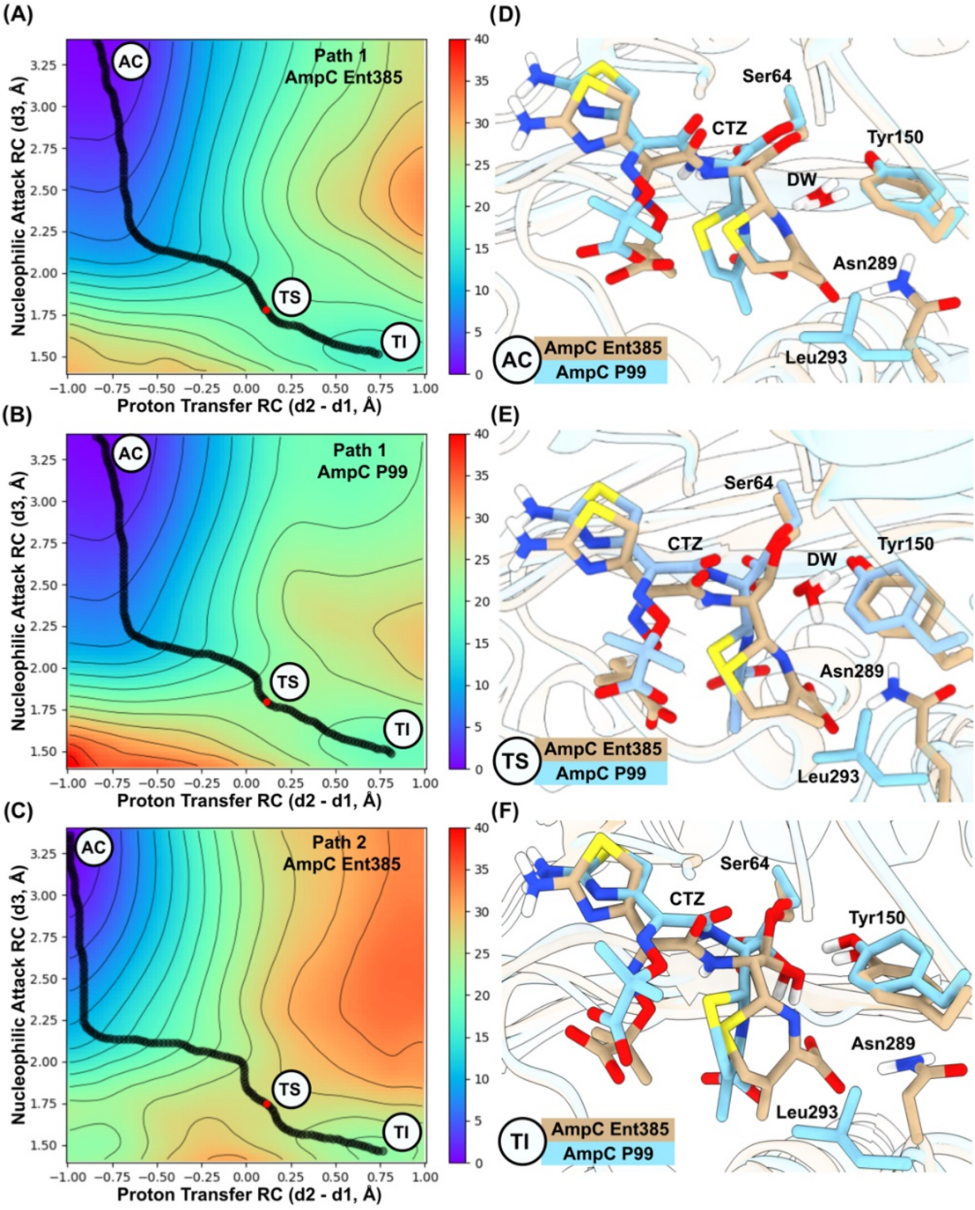
QM/MM reaction simulations of ceftazidime deacylation in Ent385 and P99 AmpC. (A–C) Two-dimensional free energy surfaces for ceftazidime hydrolysis considering the conjugate base mechanism (panels A and B) and substrate-activated mechanism (panel C), obtained from umbrella sampling at the DFTB2/ff14SB level. AC is Acyl-Enzyme, TS is the approximate Transition State (red dots), and TI is the Tetrahedral Intermediate state. The black path represents the minimum free energy path, computed using the MEPSA tool (http://bioweb.cbm.uam.es/software/MEPSA/). (D–F) Representative (as determined by clustering analysis on RMSD of ceftazidime) AC, TS, and TI structures of AmpC P99 (cyan) and AmpC Ent385 (tan). Nonpolar hydrogens have been omitted for clarity.

Next, we compared the calculated free energy barrier of Path 1 with the experimentally derived rate. Based on a *k*_cat_ of 25.8 s^–1^,^6^ and the Eyring equation, the free energy barrier (Δ^‡^ *G*) for ceftazidime hydrolysis should have an upper limit of 15.7 kcal/mol. Notably, the Δ^‡^ *G* from our initial DFTB2/ff14SB umbrella sampling of ceftazidime deacylation is higher by 3.6 kcal/mol. When performing umbrella sampling simulations with an increased QM region (now including the main chain nitrogen of Ser64, which contributes to oxyanion stabilization), this overestimation is removed, resulting in good agreement with the expected barrier derived from the experimental rate (16.0 kcal/mol; Figure S2A). Further, potential energy profile determination shows that QM/MM energies obtained with DFTB2 or hybrid-DFT (M06–2X/def2-SVP) are similar for this reaction (Figure S2C). Together, this indicates that the calculated barrier for deacylation, when corrected for the small QM region size, is consistent with deacylation representing the rate-limiting step in ceftazidime hydrolysis. Therefore, the overestimation of the barrier obtained with the smaller QM region is due to the inaccurate oxanion hole stabilization rather than shortcomings of the DFTB2 method for this particular reaction. Together, this indicates that Path 1, with Tyr150 acting as the base to abstract the proton from the DW, is consistent with experimental kinetics for ceftazidime hydrolysis in Ent385 AmpC. While DFTB2 tends to underestimate the energy barrier for deacylation in class A and D serine β-lactamases,^32,41^ this is not observed here, with Tyr150 acting as the base.

### QM/MM Simulations of Deacylation Accurately Capture the Enhanced Ceftazidime Hydrolysis Activity of AmpC Ent385

The increase in the catalytic efficiency of class C β-lactamases against extended-spectrum cephalosporins is mainly due to missense mutations that result in amino acid substitutions and/or deletions.^10^ In the particular case of AmpC Ent385, the deletion of residues 294 and 295 induces structural changes in the R2 loop (residues 286 to 310), while the key motifs ^64^SXSK, ^150^YXN, and ^315^KTG remain conserved. To elucidate the impact of these structural modifications, we performed QM/MM simulations focusing on the deacylation step. Our simulations for the native AmpC Ent385 variant yielded an activation free energy barrier of 19.3 ± 0.8 kcal/mol. To specifically assess the contribution of the Ala294–Pro295 deletion, we modeled a reinserted deletion variant (Ent385_Rev) for which the computed activation free energy barrier is 23.4 ± 0.5 kcal/mol. Experimentally, Ent385_Rev exhibits a significantly reduced *k*_cat_ of 0.03 s^–1^,^6^ corresponding to an energy difference (ΔΔ^‡^ *G*_exp_) of roughly 4 kcal/mol. The computed energy difference (ΔΔ^‡^ *G*_*calc*_) between Ent385 and Ent385_Rev is 4.1 kcal/mol, which agrees well with the experimental data.

The predicted Δ^‡^ *G* for the canonical AmpC P99 is 22.6 ± 1.0 kcal/mol, which is comparable to the value predicted for Ent385_Rev (23.4 ± 0.5 kcal/mol). Although the deletion of Ala294-Pro295 in AmpC P99 leads to an increase in rate (from 0.0061 to 0.028 s^–1^, corresponding to a ΔΔ^‡^ *G* of 0.9 kcal/mol),^42^ a direct comparison of the rates of AmpEnt385 and AmpC P99 is difficult because the *k*_cat_ values are obtained by different laboratories and protocols.

Together with the good agreement between the calculated barriers (with the larger QM region) and those inferred from the *k*_cat_ values, this indicates that deacylation is likely rate-limiting for ceftazidime hydrolysis in AmpC Ent385 and P99. This is consistent with detailed kinetic studies on several different AmpC β-lactamases, which indicate that deacylation is rate-determining for hydrolysis of ceftazidime and other third-generation cephalosporins (which are poor substrates).^11–13,43^ Tripathi and Nair previously found that acylation was more likely to be rate-limiting in the hydrolysis of the good substrate cephalotin (in 908R class C β-lactamase), but the rate-limiting step can be different for different enzyme–substrate combinations.^2,39^

The crystal structures of ceftazidime and a boronic acid transition-state analog inhibitor in complex with AmpC from suggested how ceftazidime (and other later generation cephalosporins) are not easily hydrolyzed by class C β-lactamases.^44^ Overall, their R1 moiety results in unfavorable interactions with the enzyme, particularly involving residues Val211 and Tyr221. Here, we compare the structures of the AC complexes in AmpC Ent385 and P99 by superimposing their Cα atoms. Although the structural similarity in the protein backbone was high (Cα RMSD of 0.7 Å), a notable structural difference was observed at the C4 carboxylate substituent of the β-lactam ring (Figure 2D).

Apart from the Ala294-Pro295 deletion, AmpC Ent385 also features a range of substitutions compared with AmpC P99 (Figure S3), including a substitution at position 289, where Ser in P99 is replaced by Asn in Ent385. Residue 289 is not conserved among class C β-lactamases,^10^ and Asn289 is found in several clinically relevant AmpC enzymes such as FOX-1 ()^45^ and EC-1 ().^46^ It has been indicated to play an important role in interactions with inhibitors,^47^ and thus likely can influence acyl-enzyme interactions. In AmpC Ent385, the Ala294-Pro295 deletion results in Leu293 moving away from the active site, removing a potential steric clash with the methylene group and thus providing space for Asn289 to interact with the C4 carboxylate group of ceftazidime in the AC complex. The altered interaction network compared to AmpC P99 that is facilitated by these structural changes suggests that Ent385 has adapted to better accommodate the ceftazidime acyl-enzyme, which, in turn, can be related to enhancing its hydrolytic activity for this substrate.

Examination of transition state ensembles for AmpC Ent385 compared to the “parent” P99 suggests that the Ent385 active site conformation is more conducive to deacylation, especially through the interaction of the (former) β-lactam ring nitrogen and the deacylating water (Figure S7). The resulting hydrogen bond donation by DW to this nitrogen is expected to increase the nucleophilicity of DW, similar to what was found for DW hydrogen bond donation to carbapenem acyl-enzymes (6-hydroxyethyl moiety) in OXA-48.^48^ Thus, the repositioning of residue Leu293 in AmpC Ent385 allows for better accommodation of the ceftazidime acyl-enzyme in a reactive position, which, in AmpC P99, is unlikely to occur due to steric hindrance of the methylene group with Leu293. Interaction fingerprint analysis indicates that Leu293 forms more hydrophobic interactions with ceftazidime in AmpC P99 compared with AmpC Ent385 (Figure S8). The importance of this residue was previously assessed using site-directed mutagenesis, with 15 different replacements for Leu293 tested.^49^ Only the Leu293Pro mutant (expected to lead to some restructuring of the R2 loop) exhibited increased catalytic efficiencies for cefepime and ceftazidime, likely contributing to higher MICs for these antibiotics. Interaction fingerprint analysis also highlights differences in interactions with Arg203, Asn/Ser289, and Thr316. Particularly, Asn289 in AmpC Ent385 contributes to holding the C4 carboxylate group close to the reaction center.

### Additional Oxyanion Stabilization by a Water Molecule Causes Enhanced Ceftazidime Hydrolysis in AmpC Ent385

Further analysis of the reaction simulations revealed a difference in active site hydration during deacylation between AmpC Ent385 and P99. In particular, an additional water molecule was observed to enter the oxyanion hole of the Ent385 active site (Figure 3), offering additional stabilization of the emerging oxyanion in the transition state ensemble (and beyond), thus potentially enhancing catalytic efficiency. Initially, in the AC state, this water molecule forms a bridge between Ser318-OH and the C4 substituent carboxylate group. As the reaction progresses, it then enters the oxyanion hole. This bridging water is observed in the experimentally determined structure of AmpC Ent385, whereas it is absent in that of AmpC P99 (Figure 3). In the latter, Ser318-OH and the C4 carboxylate are therefore closer together, consistent with a direct hydrogen bond. Water molecules in β-lactamases have been shown to play critical roles in catalysis and can exchange rapidly with bulk solvent. An early MD study on a class A β-lactamase already suggested that active site water molecules, including the ″hydrolytic″ and ″oxyanion hole″ waters, exchange with the bulk solvent at a rate faster than the catalytic process.^50^ In more recent work, it was shown that limiting the hydration of the general base likely plays a crucial role in determining deacylation efficiency in clinically relevant OXA-48-like β-lactamases.^22,48^

**Figure 3:**
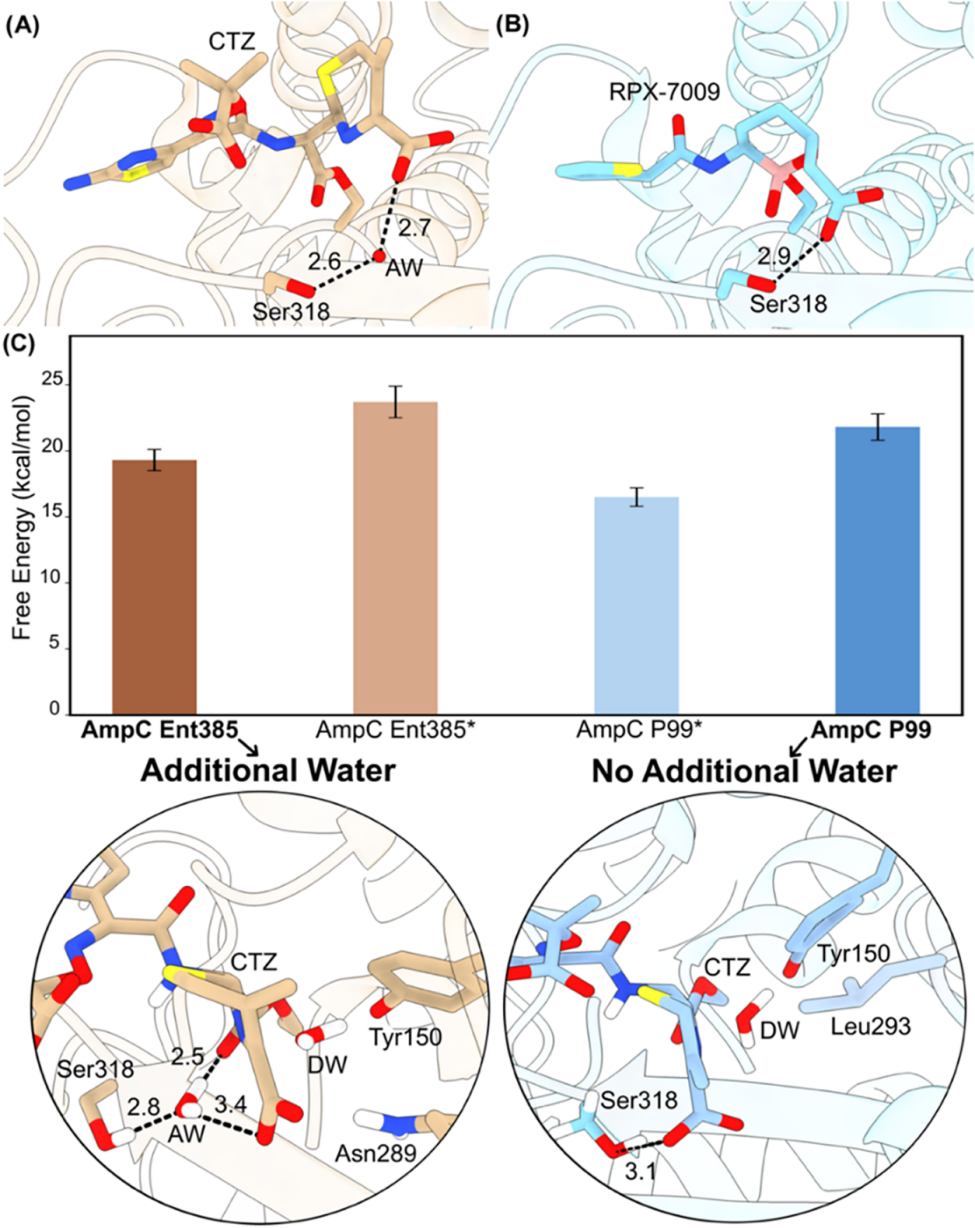
Free energy barriers and representative transition state structures from umbrella sampling simulations for AmpC Ent385 and P99 from different active site conformations. (A) Representative structure based on PDB entry 6LC9 showing ceftazidime (CTZ) bound in AmpC Ent385, highlighting the additional water molecule (AW) interactions. (B) Representative structure based on PDB entry 4XUX showing RPX-7009 bound in AmpC P99. (C) Average free energy barriers calculated from 3 (likely conformations) or 2 (unlikely conformations, marked with an asterisk) independent QM/MM umbrella sampling simulations. The lower panels illustrate detailed interactions at the transition states: left panel (AmpC Ent385) shows additional water interactions facilitated by the Ala294-Pro295 deletion; right panel (AmpC P99) shows the absence of such additional water interactions.

Here, we first investigated the difference of the interactions between Ser318 and the ceftazidime carboxylate in our MM MD simulations of the acyl-enzyme states of AmpC Ent385 and P99. We monitored the distance between the Ser318 hydroxyl oxygen and the C4 carboxylate oxygen in frames classified as “reactive conformations,” i.e., those with the distance between the oxygen of the deacylating water and the electrophilic carbon less than 3.5 Å, and with Tyr150 Oη within 2.5 Å. In AmpC P99, the Ser318-Oγ to carboxylate distance is predominantly <3 Å (as in the crystal structure), suggesting that the presence of a bridging water molecule between them is unlikely (Figure S9). Even when the distance between Ser318 and the C4 carboxylate oxygen is more than 3.5 Å, Arg349 can take up the position between them, whereas this is usually taken by the additional water molecule in AmpC Ent385 (consistent with its crystal structure). QM/MM simulations of the AC, TS, and TI states starting from the same (AC-based) enzyme conformation (where the water bridges Ser318 and the C4 carboxylate) indicate that movement of the water from the Ser318-carboxylate bridging position to the oxyanion hole occurs only when negative charge is accumulating on the oxyanion (in TS and TI), and this movement is fast (∼ps time scale, Figure S10).

To test whether the presence of the additional “oxyanion hole” water molecule in the transition state of ceftazidime deacylation by AmpC Ent385 is responsible for the lower energy barrier for deacylation, we compared reaction simulations with and without this additional water molecule in both AmpC P99 and Ent385. We identified some (rare) cases in the AmpC P99 acyl-enzyme MM MD simulations where a bridging water molecule was present. For both variants, we thus selected “reactive conformation” snapshots that included the bridging water molecule (as observed in the crystal structure of Ent385, PDB ID: 6LC9), and snapshots without this water molecule, where Ser318 directly interacted with the C4 carboxylate of ceftazidime. Each snapshot was then used as the starting point for QM/MM reaction simulations (using the same protocols used before). The average free energy barriers from these reaction simulations (Figure 3) clearly indicate that the additional water molecule, which moves to the oxyanion hole during the reaction, causes a significant reduction in the activation energy (by stabilizing the emerging oxyanion in the AmpC-ceftazidime TS complex). For AmpC Ent385, where the bridging water is expected in the acyl-enzyme (based on our MM MD simulations and the crystal structure), the reduction in barrier due to the additional water-oxyanion hydrogen bond is 4.4 kcal/mol. For AmpC P99, where no bridging water is expected, the barrier is reduced by a similar amount.

This analysis indicates that the additional water molecule is crucial for stabilizing the transition state in Ent385, thereby lowering the activation energy and enhancing the catalytic efficiency. We note that if the P99 variant was able to incorporate an additional water molecule into its active site, the resulting deacylation barrier would be significantly lower. However, both our simulation data and previous experimental findings indicate that such a conformation is unlikely to occur. Consequently, only when the additional water is present in the transition state in Ent385, and *not* in P99, does the calculated difference in barrier correctly indicate the expected difference in ceftazidime hydrolysis kinetics.

## Conclusions

Our study has unveiled the detailed mechanism underlying the enhanced hydrolysis of ceftazidime by the Ent385 variant of AmpC β-lactamase. Constant pH simulations show that Tyr150 can be present as a tyrosinate in the ceftazidime acyl-enzyme (at no free energy cost), and QM/MM umbrella sampling simulations clearly indicate that Tyr150 acts as a base in the deacylation reaction. Furthermore, these QM/MM reaction simulations capture a significant differences in Δ^‡^ *G* between the canonical AmpC (P99), the Ent385 variant and the Ent385_Rev variant for ceftazidime deacylation, which align well with the available experimental data. Transition state structure analysis revealed that the active site conformation in the Ent385 variant is better optimized for deacylation due to the Ala294-Pro295 deletion (as well as the Ser289Asn mutation). One of the consequences of this is that a water molecule can contribute to oxyanion stabilization in Ent385. Our simulations highlighted the crucial role of this extra water molecule in stabilizing the transition state in this variant, leading to increased ceftazidime hydrolysis activity. This information may aid the design of more effective inhibitors. As bacteria continually evolve and new β-lactamase variants that confer resistance emerge, the protocols employed in this study could be used successfully to capture the kinetic differences observed among other Class C β-lactamase variants with similar features, and thereby understand their detailed mechanisms. Such insights are important for developing advanced strategies to combat antibiotic resistance and improve treatment efficacy.

## Supplementary Material





## Notes

Details of molecular mechanics molecular dynamics simulations, description of QM regions in QM/MM simulations (Figure S1); free and potential energy surfaces, including DFT corrected energies (Figure S2); AmpC Ent385 vs AmpC P99 sequence alignment (Figure S3); histograms of key interaction distances (Figures S4–S5, S7 and S9), and structural representations of reaction intermediates in Path 2 (Figure S6); interaction fingerprints for ceftazidime in the AmpC P99 and Ent385 acyl-enzymes (Figure S8), and distances relevant to catalytic activity (Figure S10); root-mean-square deviation for four replicate MD simulations of three systems (Ent385, Ent385_Rev, and P99) over 120 ns (Figure S11); two-dimensional free energy surfaces for ceftazidime hydrolysis for AmpC Ent385 and P99 from different active site conformations (Figure S12); titration curves for histidine residues obtained from constant pH MD simulations in implicit solvent (Figure S13), and titration behavior of active site residues Lys67, Tyr150, and Lys315 (Figure S14) (PDF)
Simulation parameter files; input files; starting structures and representative structures from MM MD and QM/MM simulations (ZIP)
