# Supplementary material for: Insights into the Enhanced Ceftazidime Hydrolysis by Ent385 AmpC β‑Lactamase from Multiscale Simulations
Source: ACS Catal. 2025 Jun 23;15(13):11739–48. doi: 10.1021/acscatal.5c02383 (PMC12235585; doi:10.1021/acscatal.5c02383)
Supplement: Supplementary file 1 [file cs5c02383_si_001.pdf]

SUPPORTING INFORMATION

Insights into the Enhanced Ceftazidime Hydrolysis by Ent385 AmpC β-lactamase from Multiscale Simulations

Anderson H. Lima[1,2*]; Marc W. van der Kamp[1*]

[1]School of Biochemistry, University of Bristol, University Walk, Bristol BS8 1TD, United Kingdom.

[2] Laboratório de Planejamento e Desenvolvimento de Fármacos, Instituto de Ciências Exatas e Naturais, Universidade Federal do Pará, Rua Augusto Corrêa, 01, 66075-110, Belém, Pará, Brasil.

***Anderson H. Lima**

ORCID: https://orcid.org/0000-0002-8451-9912

Email: anderson@ufpa.br

***Marc W. van der Kamp**

ORCID: https://orcid.org/0000-0002-8060-3359

Email: marc.vanderkamp@bristol.ac.uk

Details of acylenzyme MM MD simulations – Energy minimization, heating, equilibration

Molecular mechanics molecular dynamics (MM MD) simulations were performed using the AMBER22 software suite. The system was prepared by solvating the structure with explicit water molecules using the TIP3P water model in tLeap. A cubic box was employed, maintaining a minimum distance of 10 Å between the protein surface and the box boundaries. Neutralising counter-ions were added (by replacing random bulk water molecules) to ensure charge neutrality. Then, a four-step energy minimisation protocol was employed to relax the system and remove steric clashes. The minimisation was conducted using the default combination of the steepest descent and conjugate gradient algorithms in AMBER. Initially, only the hydrogen atoms were minimised, with all other atoms restrained using a harmonic potential with a force constant of 100 $kcal \cdot mol^{-1} \cdot Å^{-2}$. This was followed by a minimisation of the hydrogen atoms and solvent molecules, keeping restraints on the protein heavy atoms. In the third step, the side chains and solvent were minimised while restraining the backbone atoms with a force constant of 10 $kcal \cdot mol^{-1} \cdot Å^{-2}$. Finally, the entire system was minimised without restraints, allowing for full relaxation. The steepest descent algorithm was applied during the first 2,000 cycles followed by 8,000 cycles of the conjugate gradient method for finer convergence, with the minimisation reaching a root mean square gradient of 0.001 $kcal \cdot mol^{-1} \cdot Å^{-1}$. The minimised structure was subsequently used as the starting point for four independent simulations, with each replica subjected to the same heating and equilibration protocol. The systems were gradually heated from 10 to 300 K over 200 ps under constant volume conditions. A Langevin thermostat was employed with a collision frequency of 2 ps^{-1} to

control the temperature. During this stage, positional restraints of 5 kcal·mol^{-1}·Å^{-2} were applied to the backbone atoms. Following the heating phase, the system was equilibrated in two steps. The first equilibration stage involved a 300 ps simulation under constant pressure and temperature conditions, using a Berendsen barostat to maintain a pressure of 1 atm and a target temperature of 300 K. Positional restraints of 10 kcal·mol^{-1}·Å^{-2} were applied to the protein backbone during this phase. The second equilibration stage was run for 500 ps under the same conditions but without restraints, ensuring full relaxation of the system. In both stages, temperature was controlled using the Langevin thermostat dynamics as before. After equilibration, production runs in the NPT ensemble were conducted for 120 ns, using the same thermostat and barostat settings as in equilibration To obtain four independent 120 ns trajectories for each system, heating, equilibration and production was repeated.

Details of acylenzyme Constant pH MM MD simulations

For the implicit solvent simulations, we used the the ff10 (ff99SB) force field and the mbondi2 radii set) to generate the topology and coordinate files. The system was first minimized in implicit solvent to relax any unfavorable contacts. Minimization was carried out using the Generalized Born Implicit Solvent model (igb = 2, salt concentration = 0.1 M) with positional restraints applied to the protein backbone (restraint force constant = 10 kcal·mol^{-1}·Å^{-2}). The steepest descent algorithm was applied during the first 1,000 cycles followed by 4,000 cycles of the conjugate gradient method. The system was then heated from 10 K to 300 K over 1×10^5 steps (with a 2 fs timestep) using Langevin dynamics. During heating, backbone restraints were maintained (5 kcal·mol^{-1}·Å^{-2}). The system was

equilibrated for an additional 4×10^5 MD steps at 300 K. During equilibration, the cpHMD scheme was fully enabled by setting solvph to 7.2 and by allowing protonation state change attempts every 5 MD steps. The production stage (2 ns using a 2 fs timestep) was similar to that used during equilibration. Three independent replicas were initiated from the minimized system, each with distinct initial velocity conditions.

For the explicit solvent simulations, force field parameters were assigned using the ff14SB force field for proteins and TIP3P for water molecules. The system was energy minimized for 5000 steps to remove any bad contacts. Then, the minimized structures were first subjected to a heating simulation in explicit solvent under constant volume conditions. The temperature was gradually increased from 10 K to 300 K over the first 1.6 ns, followed by an additional 0.4 ns at a constant temperature of 300 K. Afterwards, the system was equilibrated at 300 K. In the initial equilibration, the simulation was performed under constant pressure conditions to stabilize the system density. This equilibration was run for 1 ns. Subsequently, an additional equilibration was conducted in explicit solvent under constant volume conditions for 2 ns at 300 K to allow the system to further relax at the stabilized density. Production simulations (5 ns using a 2 fs timestep) were then performed at pH 7.2. Three independent replicas were initiated from the minimized system, each with distinct initial velocity conditions.

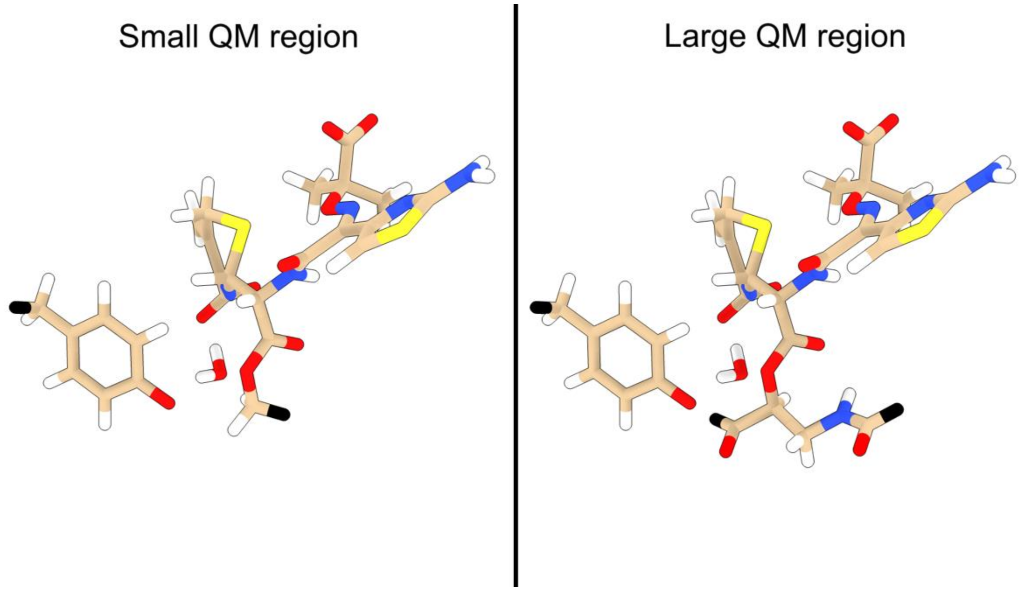

Figure S1. Representation of the QM regions in the simulations. The Small QM region includes 70 atoms and two link atoms: the full ceftazidime molecule, side chains of Ser64 and Tyr150 (starting from Cβ), and the deacylating water molecule. The Large QM region expands to include additional backbone atoms adjacent to Ser64 (83 and three link atoms).

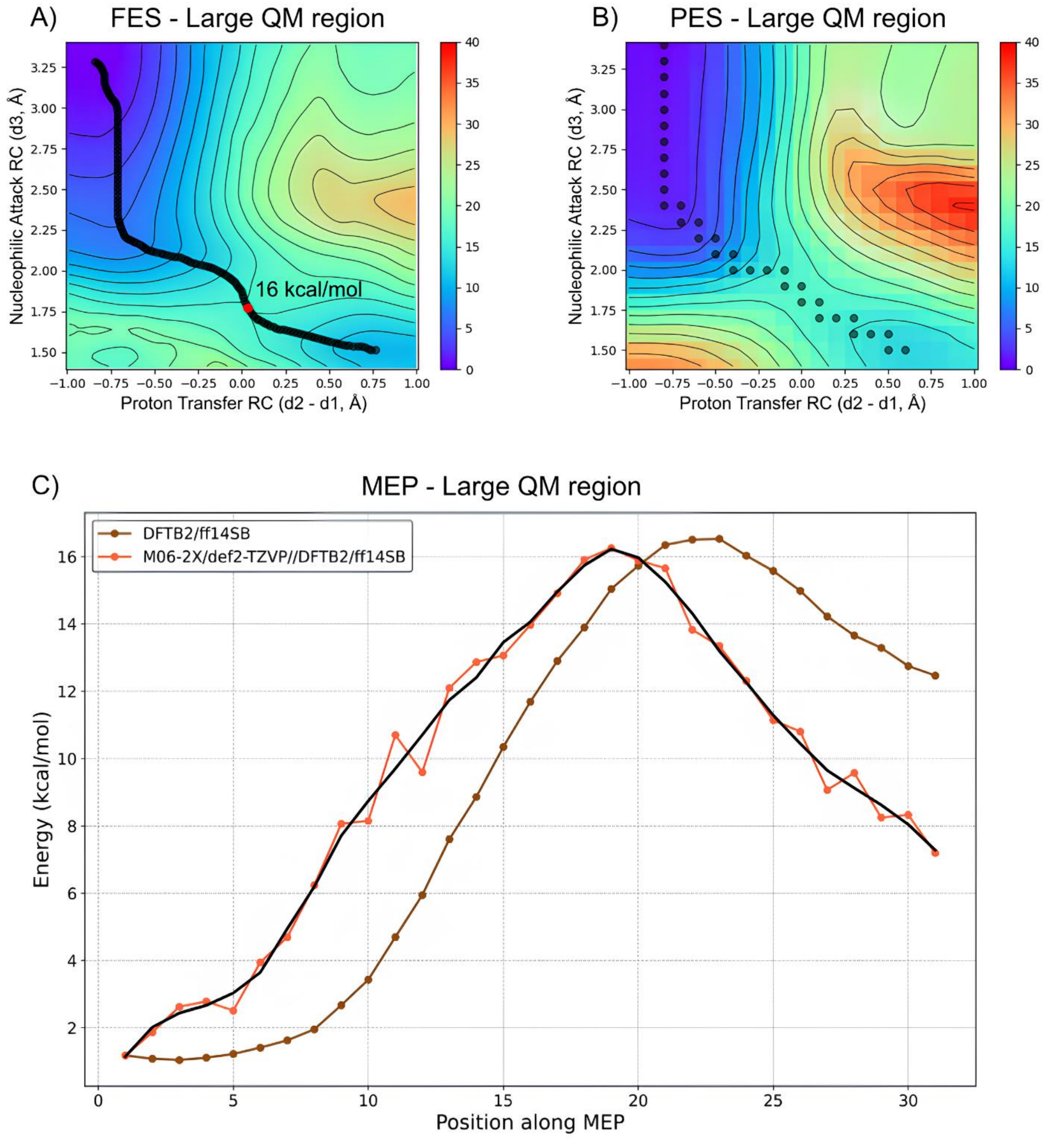

Figure S2. Testing DFTB2/ff14SB for ceftazidime hydrolysis in AmpC Ent385. A) Two-dimensional free energy surface (FES) for ceftazidime hydrolysis considering the conjugate-base mechanism (Path 1, obtained using the larger QM region, see Figure S1). The black path represents the Minimum Free Energy Path, computed using the MEPSA tool (http://bioweb.cbm.uam.es/software/MEPSA/). B) Potential energy surface (PES) for the reaction presented in A), at the same level (DFTB2/ff14SB). C) Minimum energy

pathway (MEP) extracted from B (tan line), with energies corrected using M06-2X/def2-TZVP (lighter line). The black line represents the corrected MEP fitted using a Savitzky-Golay filter. Corrections to the minimum energy path on the potential energy surface were applied using the following equation: $E_{\text{corrected}} = E_{\text{QM/MM}} - E_{\text{QM}}$ DFTB2 + E_{QM} M06-2X/def2-TZVP, where the QM/MM energies were computed using the ff14SB force field combined with the DFTB2 method. The single-point QM energies at the DFTB2 level were obtained using the SQM module within Amber and, single-point QM energies at the M06-2X/def2-TZVP level were obtained with Orca software, version 6.0 (Neese,F.; Wennmohs,F.; Becker,U.; Riplinger,C. “The ORCA quantum chemistry program package” J. Chem. Phys., 2020 152 Art. No. L224108 doi.org/10.1063/5.0004608).

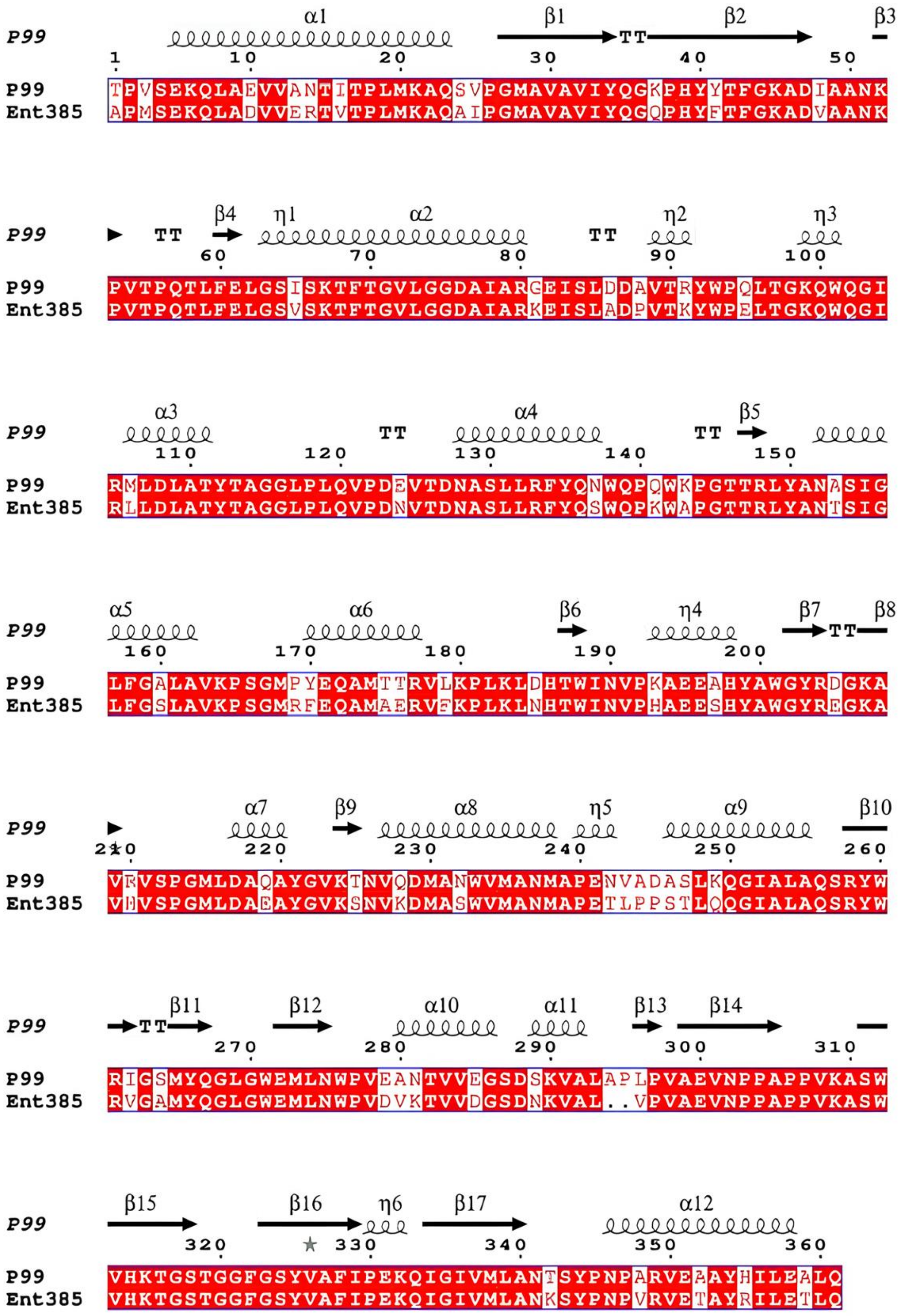

Figure S3. Sequence alignment of AmpC β-lactamases P99 and Ent385 (designed in https://espript.ibcp.fr/ESPript/ESPript/)

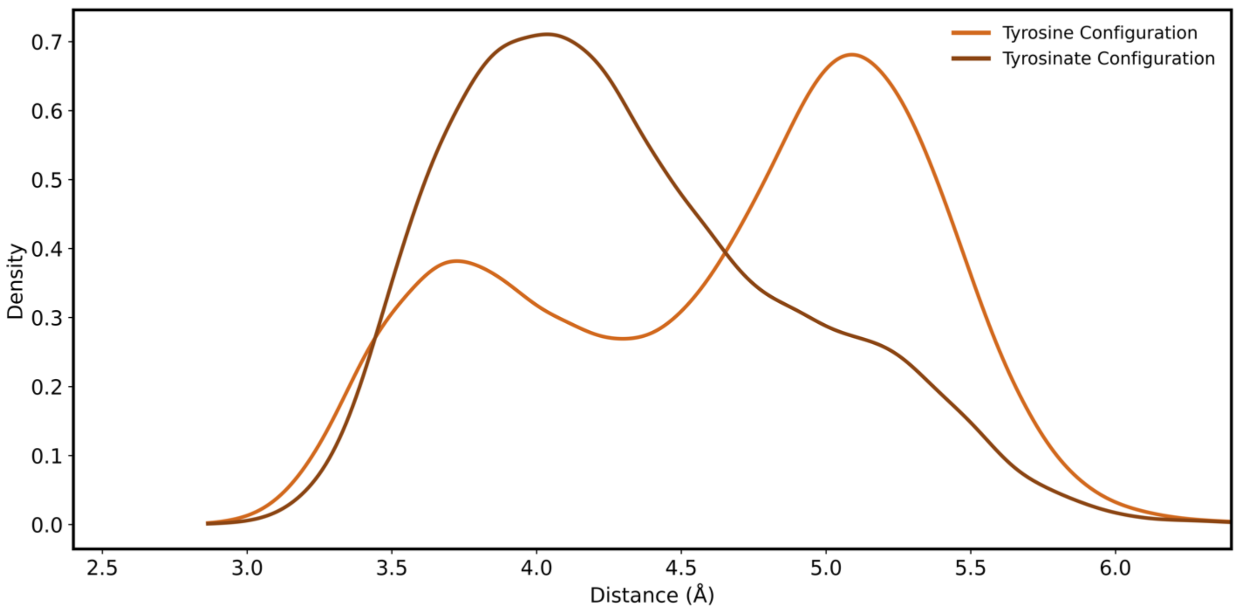

Figure S4. Kernel Density Estimate (KDE) fit of histograms of the distance between the closest solvent water and the electrophilic carbon (C8) of ceftazidime for the Tyrosine and Tyrosinate active site configurations. The Tyrosinate configuration shows a higher density around 3.5-4 Å, indicating a more favorable positioning of water for the deacylation reaction compared to the Tyrosine configuration. Data were obtained from 4 independent MD simulations of 120 ns each per system (48,000 frames per system).

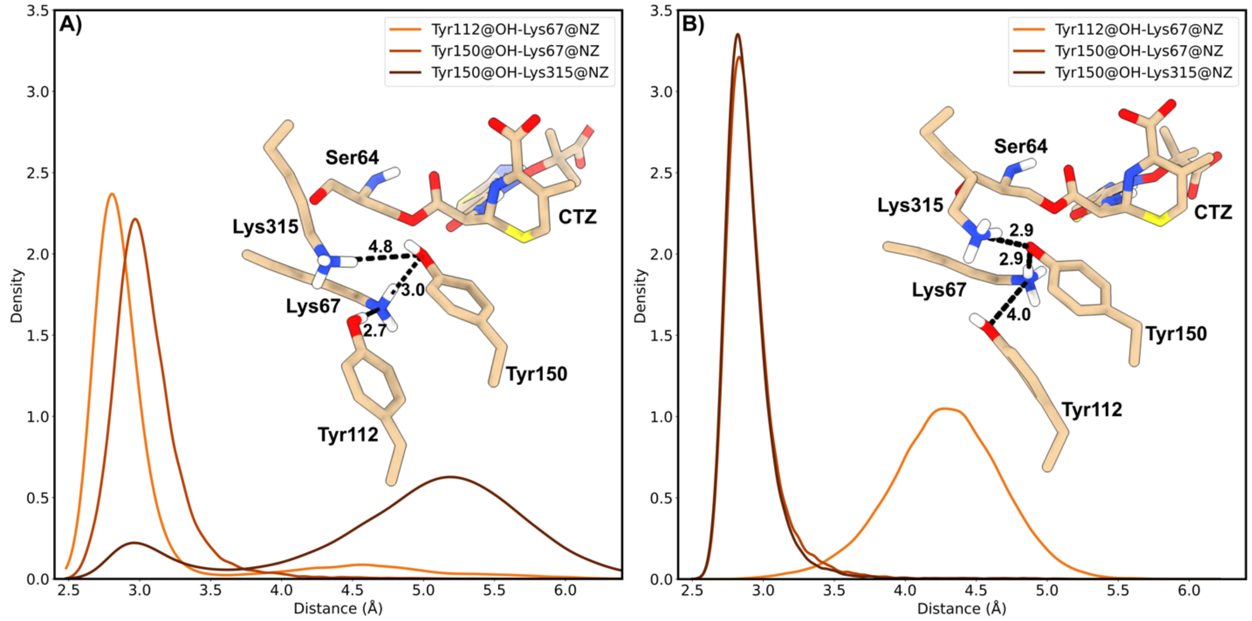

Figure S5. Kernel Density Estimate (KDE) fit of histograms of key hydrogen bond interaction distances (donor to acceptor) in the Tyrosine (A) and Tyrosinate (B) configurations, based on data from 4 independent MD simulations of 120 ns each per system (48,000 frames in total per system).

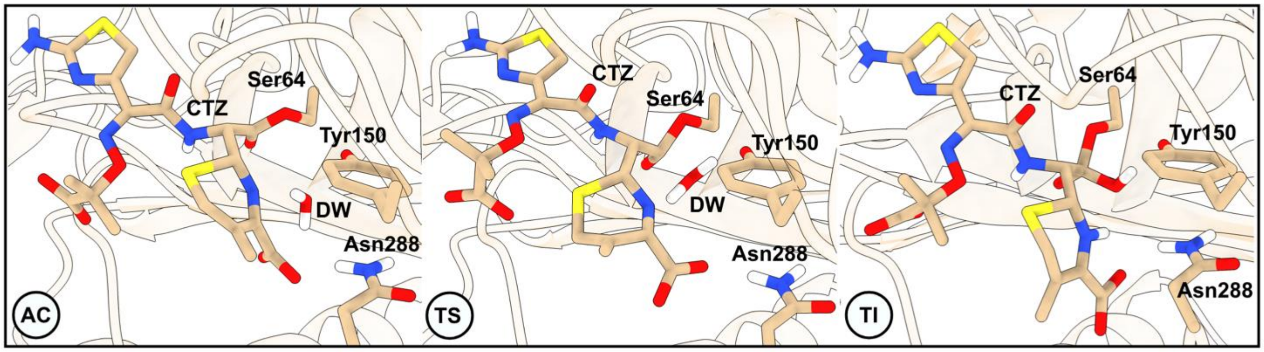

Figure S6. Representative structures of the substrate-activated mechanism (Path 2), obtained using k-means clustering on the active site RMSD. AC is Acyl-Enzyme, TS approximate Transition State, and TI the Tetrahedral Intermediate state. The non-polar hydrogens have been omitted for better clarity in visualization.

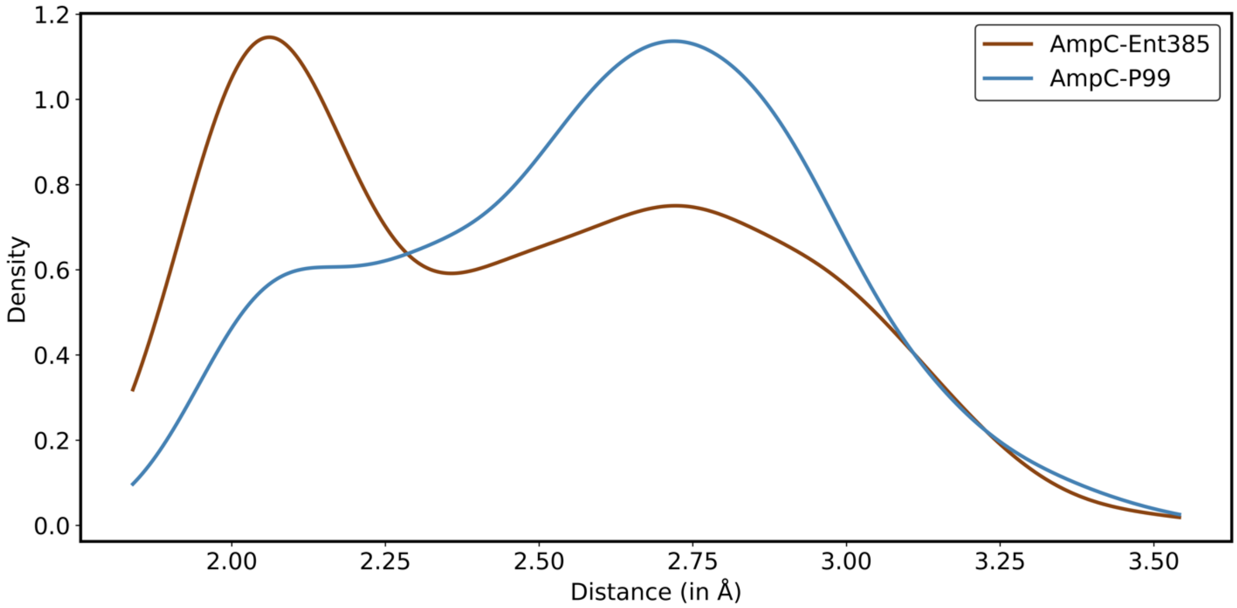

Figure S7. Kernel Density Estimate (KDE) fit of histograms for the interaction between the β-lactam ring nitrogen and the closest DW hydrogen over 100 ps of the TS QM/MM MD (umbrella sampling window RC_1=0.1, RC_2=1.8).

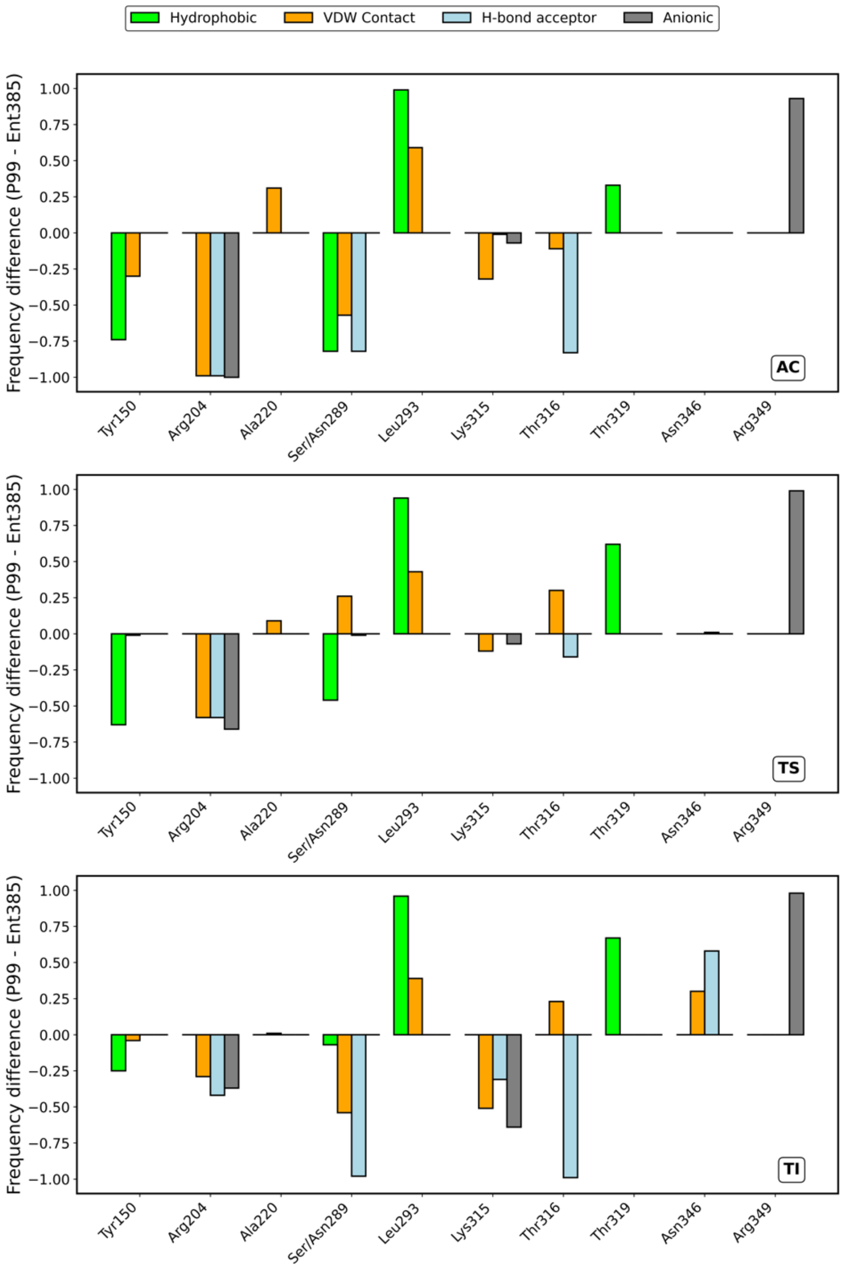

Figure S8. Interaction fingerprint for ceftazidime with AmpC P99 and Ent385 acyl-enzyme (AC), transition state (TS) and tetrahedral intermediate (TI) ensembles. For each state, the fingerprint quantifies the frequency of key non-covalent interactions (e.g., hydrophobic, van der Waals contacts, H-bond acceptor and anionic) between ceftazidime and active site residues. Frequency differences (expressed as fractions ranging from 0 to 1) are plotted such that positive values indicate an interaction is more frequently observed in AmpC P99, whereas negative values indicate a preference for AmpC Ent385.

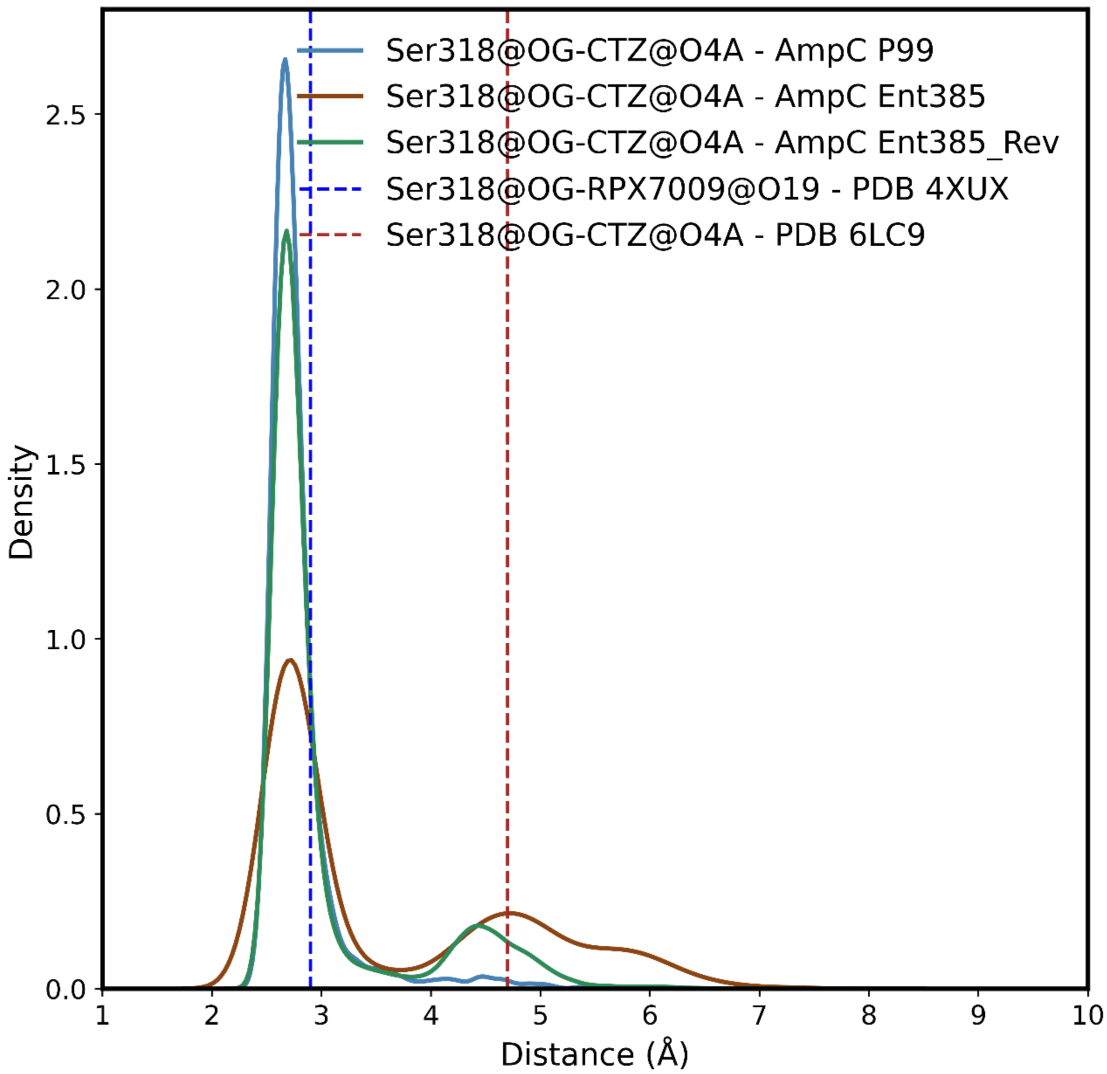

Figure S9. Kernel Density Estimate (KDE) fit of histograms for the interaction between the Ser318 OG atom and ceftazidime O4A atom (C4 carboxylate group) in 'reactive' snapshots (distance between the DW oxygen and electrophilic carbon < 3.5 Å, and with Tyr150 Oη < 2.5 Å) from MM MD simulations. Dashed lines indicate Ser318 OG atom distances to ceftazidime O4A atom (blue) or RPX7009 O19 atom (tan) in corresponding crystal structures (PDB IDs 6LC9 and 4XUX, respectively).

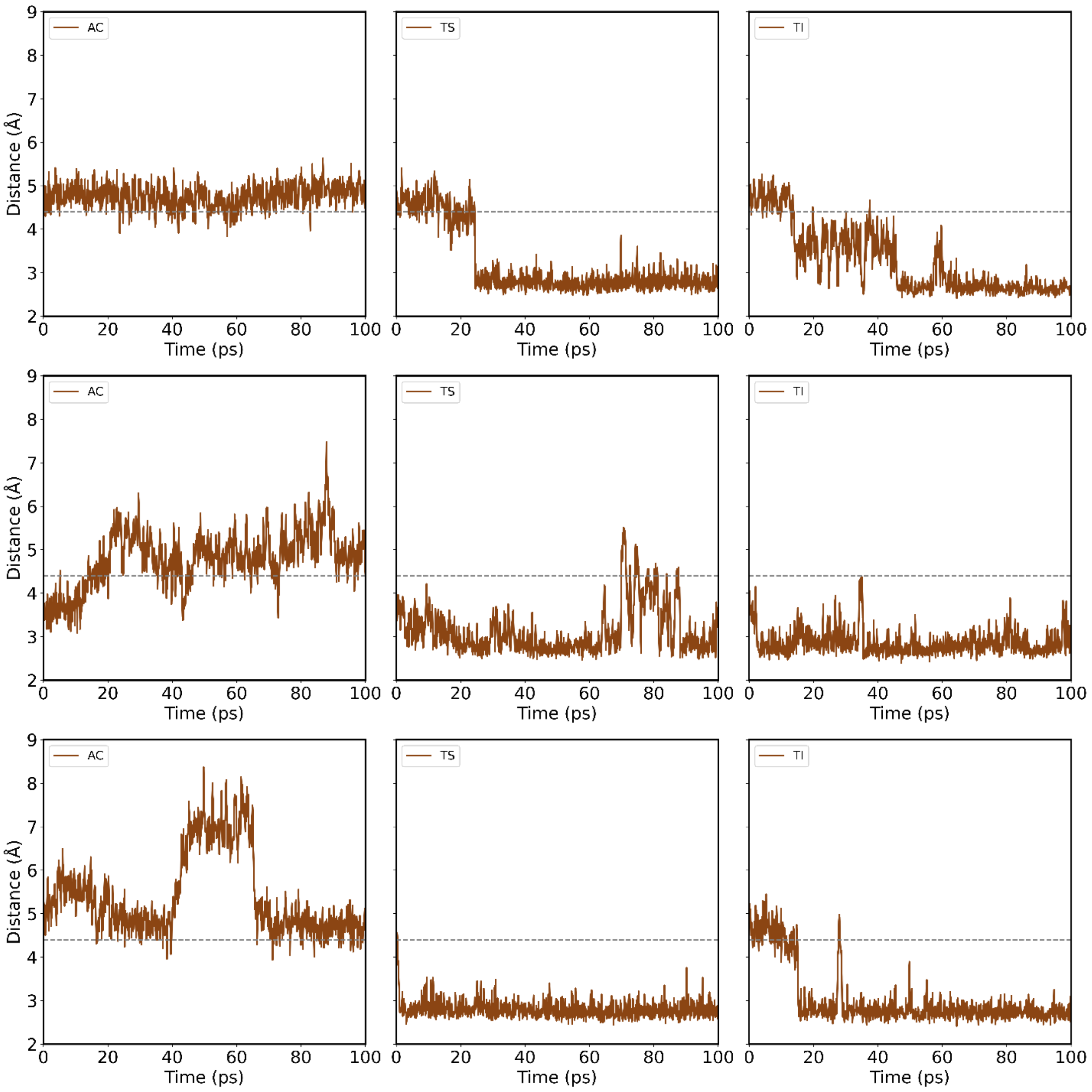

Figure S10. Distances between the oxyanion hole oxygen and the additional water molecule over 100 ps in the AC, TS and TI QM/MM MD replicas for AmpC Ent385 (A-C). The dashed lines indicate the corresponding distance in the AmpC Ent385 crystal structure (PDB ID 6LC9, HOH 533). First, to ensure structural consistency around the active site and generate comparable enzyme-substrate conformations, where the additional water molecule had not already moved during previous umbrella sampling windows, the TS and TI ensembles were created by performing a minimization of the endpoint of the AC umbrella sampling window, applying reaction coordinate restraints corresponding to the TS (RC_1=0.1, RC_2=1.8 Å).

The system was then gradually heated (from 10 to 300 K over 5 ps) and 100 ps QM/MM MD was performed with the TS or TI window umbrella sampling restraints. During this simulation, the water molecule enters the active site and then forms a hydrogen bond with the (emerging) oxyanion.

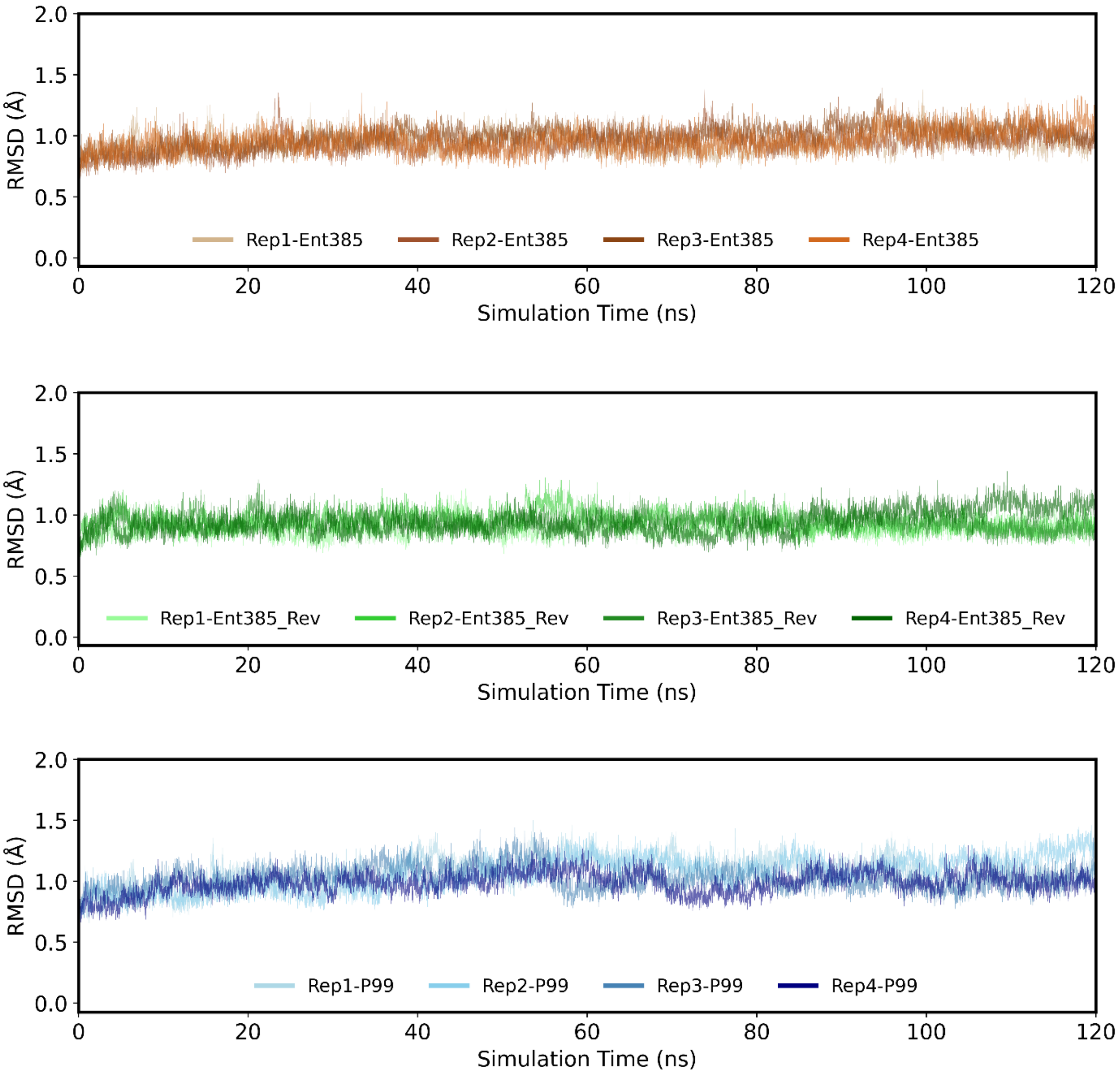

Figure S11. The root-mean-square deviation (RMSD) for four replicate molecular dynamics simulations of three systems (Ent385, Ent385_Rev, and P99) over 120 ns. The top panel shows RMSD traces for Ent385 (Rep1–Rep4), the middle panel for Ent385_Rev (Rep1–Rep4), and the bottom panel for P99 (Rep1–Rep4). RMSD was calculated using the Cα atoms of the protein.

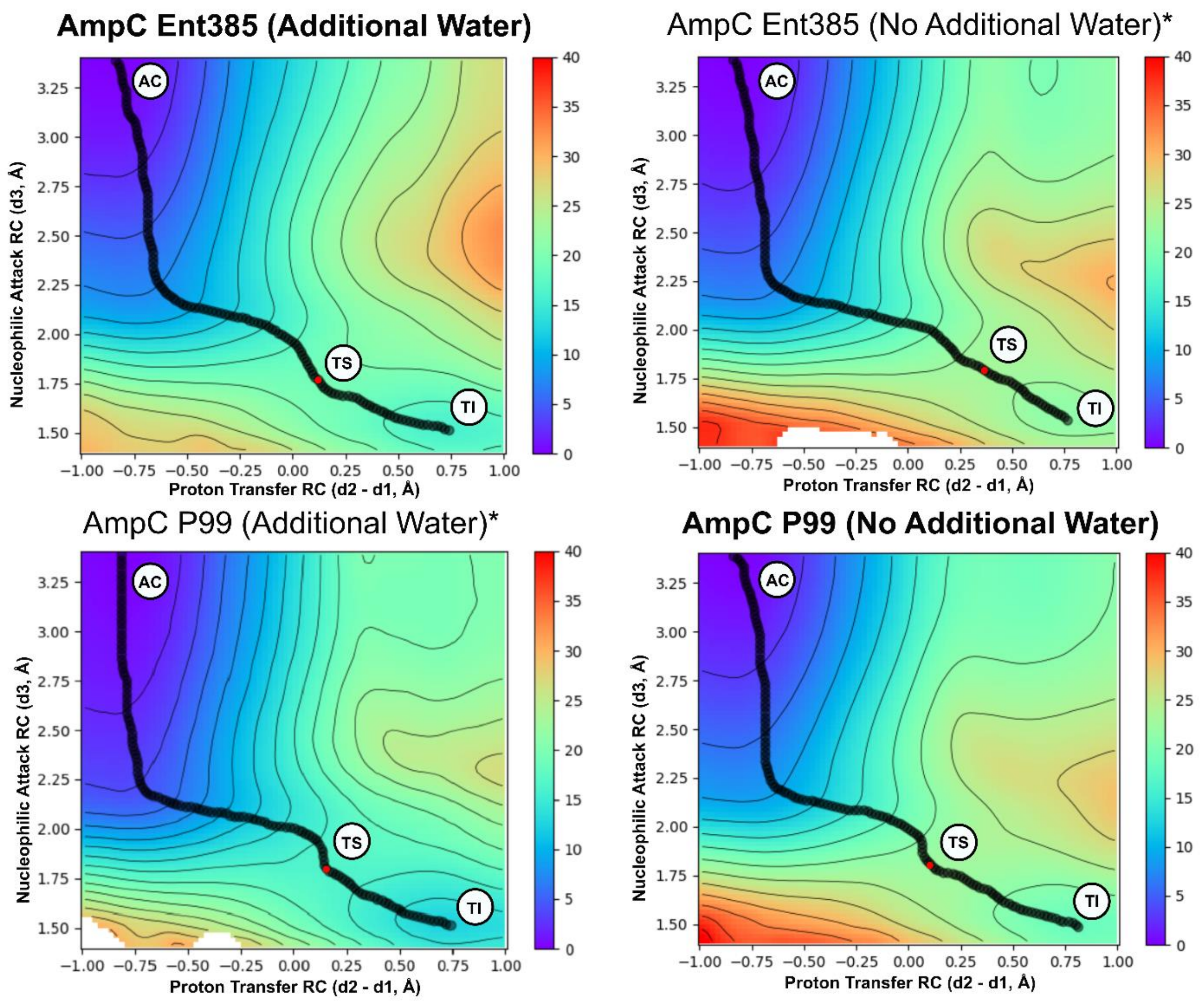

Figure S12. Two-dimensional free energy surfaces for ceftazidime hydrolysis considering AmpC Ent385 and P99 from different active site conformations. AC is Acyl-Enzyme, TS the approximate transition state (red dots), and TI the Tetrahedral Intermediate state. The black path represents the Minimum Free Energy Path, computed using the MEPSA tool (http://bioweb.cbm.uam.es/software/MEPSA/). *Represents unlikely conformations of each variant.

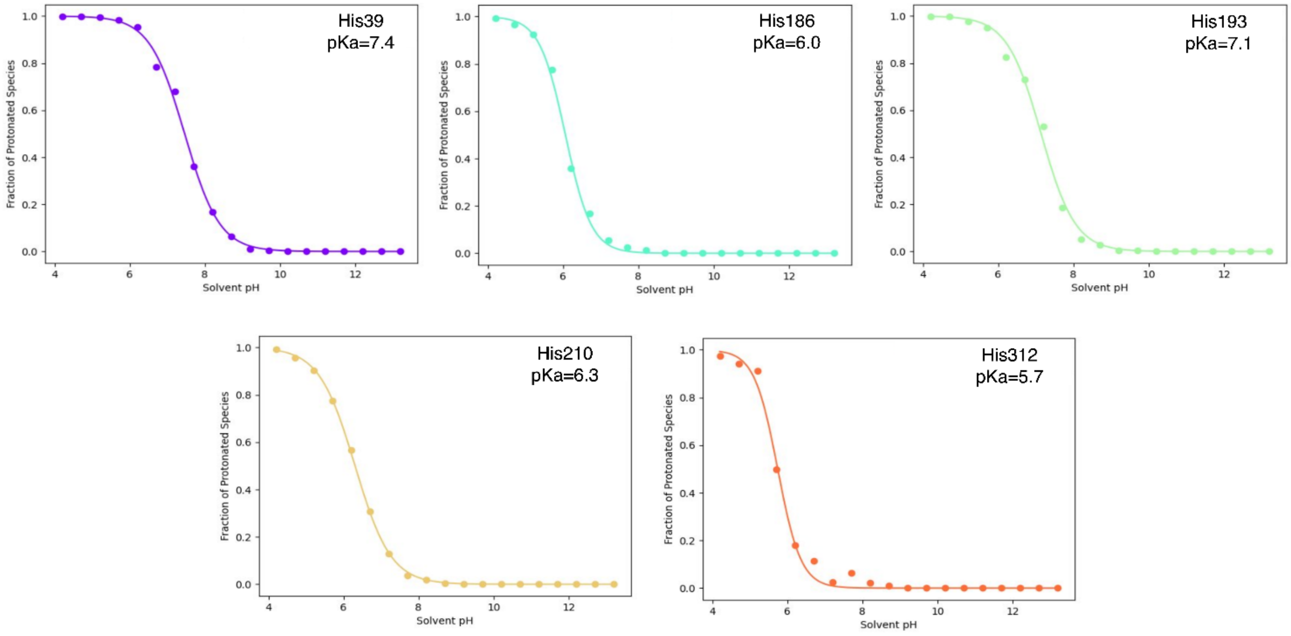

Figure S13. Titration curves of histidine residues obtained from constant pH molecular dynamics simulations in implicit solvent. Protonation fractions were determined across a pH range from 4.2 to 13.2 and fitted using the Hill equation to estimate pKa values.

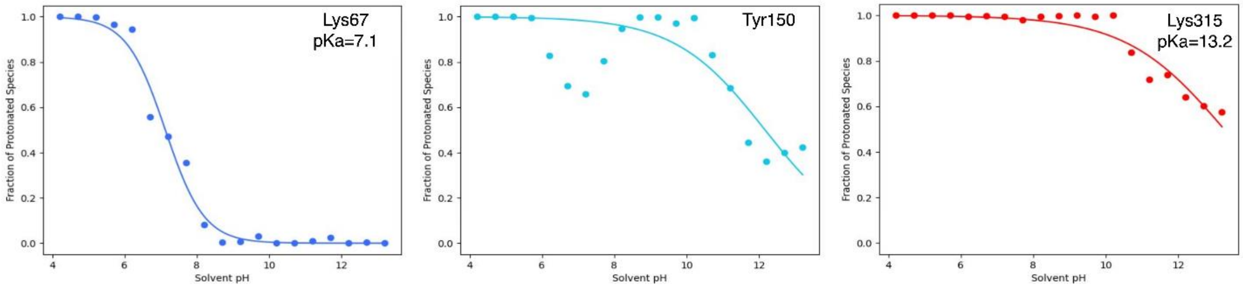

Figure S14. Titration curves of Lys67, Tyr150, and Lys315 from constant pH molecular dynamics simulations in implicit solvent.
